# Kinetics of single and dual simultaneous infection of pigs with swine influenza A virus and porcine reproductive and respiratory syndrome virus

**DOI:** 10.1111/jvim.15832

**Published:** 2020-07-03

**Authors:** Małgorzata Pomorska‐Mól, Katarzyna Podgórska, Ewelina Czyżewska‐Dors, Hanna Turlewicz‐Podbielska, Maciej Gogulski, Jan Włodarek, Anna Łukomska

**Affiliations:** ^1^ Department of Preclinical Sciences and Infectious Diseases, Faculty of Veterinary Medicine and Animal Sciences Poznan University of Life Sciences Poznań Poland; ^2^ Department of Swine Diseases National Veterinary Research Institute Pulawy Poland

**Keywords:** coinfection, disease, immune and acute phase response, viral load, viral shedding

## Abstract

**Background:**

Simultaneous viral infections exhibit the phenomenon of viral interference, but understanding of the effect of one virus on another is limited.

**Objective:**

Evaluate and compare clinical characteristics, immune and acute phase response, viral shedding and viral load in pigs singly and doubly inoculated with swine influenza A virus (swIAV) and porcine reproductive and respiratory syndrome virus (PRRSV).

**Animals:**

Fifty‐four 7‐week‐old piglets.

**Methods:**

Clinical status and gross lung lesions were scored. Titration of swIAV was carried out in Madin‐Darby canine kidney cells. The PRRSV RNA was quantified using a commercial qPCR kit. Antibodies were detected by hemagglutination inhibition assay and commercial ELISA. A lymphocyte proliferation assay was used to measure antigen‐specific T‐cell responses. Acute phase proteins were determined using ELISA.

**Results:**

No differences were found between mean clinical scores, swIAV and PRRSV shedding, and magnitude of the humoral and T‐cell response between single‐inoculated and dual‐inoculated groups. Concentrations of C‐reactive protein and haptoglobin increased in PRRSV‐inoculated and coinoculated groups, whereas serum amyloid A concentration was increased in groups inoculated or coinoculated with swIAV. Mean swIAV TCID_50_ titers in the lungs did not differ significantly between coinoculated and swIAV single‐inoculated pigs. A significantly higher mean copy number of PRRSV was found in the lungs of PRRSV only‐inoculated pigs at 2 day postinoculation (DPI). From 4 DPI, no significant differences in PRRSV load were identified.

**Conclusions and Clinical Importance:**

Coinfection of pigs with swIAV and PRRSV did not potentiate clinical signs, lung lesions, immune response, and replication of the viruses in the respiratory tract.

**Abbreviations**
AUC: area under the curve
CRP: C‐reactive protein
DPI: day postinoculation
HI: hemagglutination inhibition
Hp: haptoglobin
LS: lung score
MDCK: Madin‐Darby canine kidney
OD: optical density
ORF: open reading frame
PBMC: peripheral blood mononuclear cells
PBS: phosphate‐buffered saline
Pig‐MAP: pig major acute phase protein
PRDC: porcine respiratory disease complex
PRRSV: porcine reproductive and respiratory syndrome virus
RSV: respiratory syncytial virus
SAA: serum amyloid A
SIV: swine influenza virus
swIAV: swine influenza virus
TCID50: 50% tissue culture infective dose

## 1 INTRODUCTION

Several respiratory viruses can participate in simultaneous infections in humans and animals, including pigs.^1^, ^2^, ^3^, ^4^, ^5^, ^6^, ^7^ Concurrent infections with several respiratory viruses, including swine influenza A virus (swIAV), porcine reproductive and respiratory syndrome virus (PRRSV), porcine circovirus type 2 or porcine respiratory coronavirus have been reported.^6^, ^7^, ^8^ Moreover, PRRSV and swIAV, together and individually, frequently are primary or secondary agents responsible for porcine respiratory disease complex (PRDC).^9^, ^10^

Simultaneous viral infections can exhibit viral interference in which 1 virus blocks the growth of another virus.^11^ Because mixed respiratory tract infections often are observed in animals, including pigs, the effect of the interaction of pathogens on the course of infection warrants further study. The impact of the intensity of coinfection on severity and clinical outcome still is unclear. Some studies determined that the clinical outcome of viral coinfections may be less, or at least not more, severe than infection by a single virus.^2^, ^6^, ^7^, ^12^, ^13^ In contrast, other studies found that viral coinfection exacerbated the clinical course.^1^, ^13^ Contradictory consequences of viral coinfections also have been reported in the human medical literature.^14^, ^15^, ^16^

Coinfections with swIAV and PRRSV are common in pig herds.^17^, ^18^, ^19^ Both PRRSV and swIAV are responsible for PRDC, and some studies indicate the possibility of synergistic effects.^6^, ^20^ Because many PRRSV strains may have immunosuppressive potential, they may impact the immune response against other pathogens.^20^, ^21^ Previous studies showed various clinical outcomes with dual PRRSV and swIAV infection.^6^, ^7^, ^20^ No significant changes in the clinical course of infection were found in a study in which piglets were infected with PRRSV and 1 week later infected with swIAV.^7^ In contrast, another study reported more severe disease after dual infection compared to single PRRSV infection.^20^ In yet another study of PRRSV and swIAV, variable clinical outcomes were observed in pigs coinfected with PRRSV and swIAV.^6^

Regardless of the results of previous coinfection studies, our understanding of the effect of 1 virus on the other at both clinical and cellular levels still is limited. Thus, our objective was to assess and compare clinical characteristics, immune and acute phase response, viral shedding, and viral load between pigs singly and doubly inoculated with swIAV and PRRSV.

## 2 MATERIALS AND METHODS

### 2.1 Viruses

The swIAV used in our study, an avian‐like H1N1 A/Poland/Swine/14131/2014 virus (SwH1N1), had been isolated from a pig suffering from acute swine influenza. The stock used for inoculation represented the third passage in eggs. The virus concentration was evaluated in a Madin‐Darby canine kidney (MDCK) cell line.

The PRRSV strain PL15‐33 was isolated from lung tissue obtained from a pig with respiratory clinical disorders. Sequencing of open reading frame (ORF) 5 and ORF7 fragments indicated that the strain belonged to subtype 1 of PRRSV‐1 and the levels of nucleotide identity compared to the prototype strain Lelystad were 88.1% and 90.9%, respectively. The strain was isolated in a primary cell culture of porcine alveolar macrophages obtained from Danish Technical University, National Veterinary Institute. An isolate was titrated in macrophages cultured in 96‐well plates after a third passage.

### 2.2 Experimental design

Fifty‐four 7‐week‐old conventional piglets from an influenza‐ and PRRS‐negative farm were used. Pigs at the sourced farm were seronegative for pseudorabies virus and *Mycoplasma hyopneumoniae*. No evidence of streptococcosis or atrophic rhinitis was found based on clinical, serological and pathological examinations. Piglets were allocated randomly to 4 groups (PRRSV [n = 14]; swIAV + PRRSV [n = 14]; swIAV [n = 14]; control [n = 12]). An equal number of gilts and boars were included in each group. Before the start of the study, all experimental animals were free of influenza A and PRRS viruses and antibodies as determined by hemagglutination inhibition assays using A/Poland/Swine/14131/2014 (H1N1), A/swine/England/96 (H1N2), A/swine/Flanders/1/98 (H3N2) and pdm‐like H1N1 (A/swine/Poland/031951/12); commercial ELISA (VetExpert PRRS Ab ELISA 4.0 BioNote, Korea) and PCR tests (for swIAV according to procedure a previously described,^22^ and for PRRSV using a commercial test according to the manufacturer's recommendation (EZ‐PRRSV MPX 4.0 real‐time PCR kit, Tetracore, USA).

During the study, animals were housed in a biosafety level 3 animal facility in independent units. Animal use and handling protocols were approved by 2nd Local Ethical Commission for Animal Experiments of University of Life Sciences in Lublin (number of approval: 77/2014).

On day 0, piglets from swIAV and swIAV + PRRSV groups were inoculated intranasally (IN) with SwH1N1 (10^7^ 50% tissue culture infection doses [TCID_50_] in 2 mL of phosphate‐buffered saline (PBS). Piglets from PRRSV and swIAV + PRRSV groups were inoculated IN with PRRSV (10^5^ TCID_50_ in 2 mL of PBS). For coinoculated pigs, the inoculum was mixed just before IN administration. The final volume of inoculum for this group was the same (2 mL per pig). Twelve pigs mock‐inoculated with PBS (2 mL) served as controls.

### 2.3 Clinical and pathological examination

Animals were examined daily from day 7 preinoculation until the end of the experiment at day 21 DPI or until euthanasia (at 2, 4, and 10 DPI). The pigs were observed and scored for respiratory signs as follows: respiratory rate: 0—normal, 0.33—slightly increased, 0.66—moderately increased, slight abdominal breathing, 1—clearly increased, distinct abdominal breathing; nasal discharge: 0—absent, 1 present; coughing: 0—absent, 1 present; sneezing: 0—absent, 1 present, anorexia: 0—absent, 1 present. Rectal temperature was measured daily. Fever was recorded when rectal temperature reached or exceeded 40°C. When long‐term fever (at least 3 days) was observed an additional point was added to the clinical score. Scores determined in each category were summated for a total clinical score for each individual pig (0‐6). Nasal swabs were collected daily from all animals. Blood samples were collected at −7 days, day 0 (inoculation), and 1, 2, 3, 5, 7, 10, 14, and 21 DPI. Three piglets of the inoculated and control groups were euthanized at 2, 4, and 10 DPI. The remaining inoculated pigs were euthanized and necropsied at 21 DPI.

#### 2.3.1 Lung score

Gross lung lesions were used to assign a lung score (LS) as described previously.^23^ Each lung lobe was assigned a number reflecting an approximate volume percentage of the entire lung represented by that lobe. Ten possible points (5 for dorsal, 5 for ventral) were assigned each to the right anterior lobe, right middle lobe, anterior part of the left anterior lobe, and caudal part of the left anterior lobe. The accessory lobe was assigned 5 points, and 27.5 points (15 for dorsal and 12.5 for ventral) were assigned to each of the right and left caudal lobes for a total of 100 points. The evaluation based on this procedure resulted in a LS that corresponded to the percentage of the lung affected by pneumonia.

### 2.4 Laboratory examinations

#### 2.4.1 Virological examination of swabs and tissue samples

Virus titration (SwH1N1) of nasal swabs and lung homogenates was carried out in MDCK cells.^24^ Homogenates (10% wt/vol) were prepared by suspending 2‐3 g of samples of lung in an appropriate volume of Dulbecco's Modified Eagle Medium (DMEM) supplemented with 1% antibiotic‐antimycotic solution (Sigma Aldrich, USA) and homogenization using an homogenizer X620 (CAT, Germany). Clarified material was stored at −80°C until virus titration. Serial 10‐fold dilutions of nasal swabs and 10% homogenates of lung were prepared in DMEM. The MDCK cells cultured in 96‐well plates were inoculated and examined for cytopathic effects after 48 to 72 hours of incubation at 37°C. The detection limit was equal 1.7 TCID_50_. Virus titers were calculated as previously described.^25^

The PRRSV RNA was isolated from 140 μL of serum, 10% lung tissue homogenate or nasal swab suspension based on QIAamp Viral RNA MiniKit (Qiagen, USA). The RNA was eluted in 60 μL of elution buffer and stored at −80°C until analysis. The PRRSV RNA was detected and quantified using EZ‐PRRSV MPX 4.0 real‐time PCR kit (Tetracore, USA) and Mx3005P system (Stratagene, USA). Each reaction was performed in a 12.5 μL volume (9 μL of reaction mix and 3.5 μL of RNA). European (EU) PRRSV Quantification Standards of known copy numbers (10^2^‐10^5^ copies/μL; Tetracore, USA) were used to construct a standard curve. The temperature profile included 15 minutes at 48°C (reverse transcription), 2 minutes of initial denaturation at 95°C, 40 cycles of denaturation (95°C, 5 seconds), and primer annealing and elongation (60°C, 40 seconds). The results were expressed as copy number/ml of serum (or nasal swab suspension) and copy number/g of tissue. The analytical sensitivity of reaction reached 4 copies of viral RNA per reaction. The reaction was linear within a 10^1^‐10^5^ copies/reaction range.

#### 2.4.2 Serological tests

All sera were examined using a hemagglutination inhibition (HI) assay against challenge SwH1N1 strain and ELISA (VetExpert PRRS Ab ELISA 4.0; BioNote, Korea). The HI assay was performed according to the standard procedure,^26^ using 0.5% chicken erythrocytes and 4 hemagglutinating units of SwH1N1. Additionally, to evaluate the immune status of the pigs before inoculation, the HI assay also was performed using H3N2 (A/sw/Ghent/172/2008, kindly provided by Laboratory of Virology, Faculty of Veterinary Medicine, Ghent University), A/sw/Poland/KPR9/2004 (isolated from the lung of a pig with influenza), and H1N2 (A/Sw/Granstedt/2004, kindly provided by IDT Biologika, Germany). All sera were tested in serial 2‐fold dilutions, starting at 1:20. For estimates of antibody concentration, titers ≥20 were considered positive.

The ELISA assays for PRRSV‐specific antibodies were conducted according to manufacturer's recommendations.

#### 2.4.3 Lymphocyte proliferation assay

The T‐cell proliferation assay to measure SwH1N1 and PRRSV‐specific T‐cell responses of pigs was performed at 0, 7, 14 and 21 DPI, as described previously.^27^ Briefly, peripheral blood mononuclear cells (PBMC) were isolated from blood samples by centrifugation on Histopaque 1.077 (Sigma, USA) and were washed twice with PBS. The isolated PBMC were seeded in plastic vials at a density of 1 × 10^6^ viable cells per vial in 1 mL Roswell Park Memorial Institute Medium (RPMI) 1640 containing 10% fetal bovine serum, 2 mM l‐glutamine, and 1% antibiotic‐antimycotic solution. For analysis of cellular responses, PBMC were restimulated in vitro with 50 μL of medium containing live SwH1N1 virus (titer 10^6.5^ TCID_50_/50 μL) or live PRRSV (10^5^ TCID_50_). In control vials, the cells were incubated without the virus (mock control) or with 5 μg/mL of concanavalin A (Con‐A; vitality control). All samples were analyzed in triplicate.

After 72 hours of incubation at 37°C in 5% CO_2_ atmosphere, the cultures were pulsed with 0.5 μCi [3H]‐thymidine (MP Biomedicals, USA). After 18 hours of incubation, the cells were harvested and the radioactivity incorporated was measured in a liquid scintillation counter (Tri‐Carb 2500TR, Packard, USA). Proliferation was expressed as stimulation index (SIx) calculated as the number of counts per minute (cpm) for virus‐stimulated cells, divided by the number of cpm for the mock‐stimulated cells (in each case taking the mean value of triplicate vials). Based on the SIx values of the control group (mean plus 3 × SD), an SIx value >4.32 or 3.80 was considered positive for PRRSV or SwH1N1, respectively.

#### 2.4.4 Acute phase proteins

The 4 acute phase proteins (C‐reactive protein [CRP], haptoglobin [Hp], serum amyloid A [SAA], and pig major acute phase protein [Pig‐MAP]) were examined using commercial assays according to the manufacturers' recommendations (Pig C‐reactive protein ELISA and Pig haptoglobin ELISA, Life Diagnostics, USA; Pig‐MAP KIT ELISA, Acuvet Biotech S.L., Spain; Phase Serum Amyloid A Assay, Tridelta Development Ltd County Kildare, Ireland). The quantity of the protein was calculated based on the standard curve for each protein using FindGraph software.

### 2.5 Statistical analysis

Data were subjected to the Shapiro‐Wilk test for normality and Levene's test for equality of variances. Nasal shedding (expressed as area under the curve [AUC]), clinical scores, and lung scores were compared between groups using a nonparametric Kruskal‐Wallis test with post hoc multiple comparisons for comparison of all pairs. The nonparametric Friedman test was used to compare observations repeated on the same subjects (acute phase protein concentrations). The lung viral load and concentrations of acute phase proteins in serum at each time point were compared between groups using ANOVA followed by Tukey's post hoc test. Differences were considered significant at *α* < .05. All calculations were performed using Statistica 13.0 (Statsoft, Poland).

## 3 RESULTS

### 3.1 Clinical outcome

**FIGURE 1:**
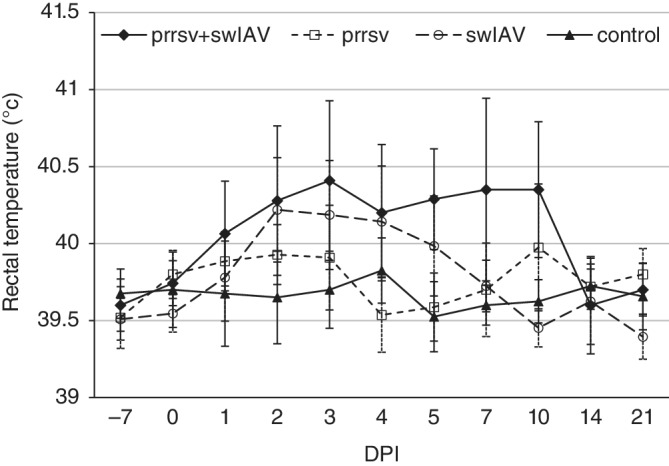
Rectal temperature (mean ± SD) in pigs singly or coinoculated with swine influenza A virus (swIAV) or porcine reproductive and respiratory syndrome virus (PRRSV) or both

Seven of 14 (50%) piglets inoculated with SwH1N1 and 3 of 14 (21.5%) inoculated with PRRSV had persistent fever (>3 days) reaching 40.0°C‐41.4°C. In the coinoculated group, persistent fever was observed in 10 of 14 piglets (71.5%; Figure 1). Moreover, in coinoculated piglets, fever was observed longer (up to 10 DPI) compared to other inoculated groups. In the swIAV group, fever peaked at 2 DPI, whereas in coinoculated pigs 2 peaks were observed (at 3 and 7‐10 DPI). In the PRRSV group similar kinetics of rectal temperature were noted (2 peaks) as in the coinoculated group.

**FIGURE 2:**
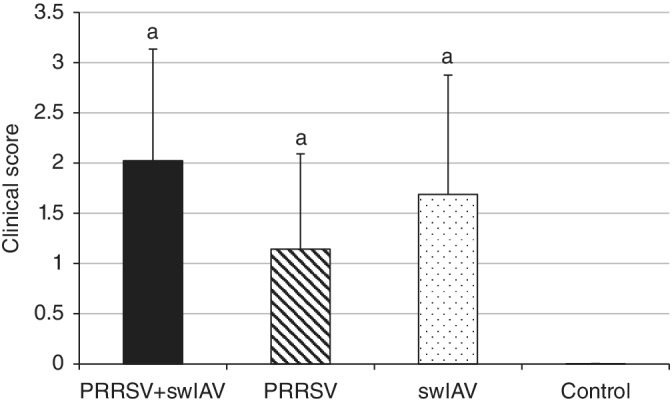
Clinical score (mean ± SD) of pigs singly or coinoculated with swine influenza A virus (swIAV) or porcine reproductive and respiratory syndrome virus (PRRSV) or both. a, significant difference with respect to controls (*P* < .05)

No significant differences were found between mean clinical scores in the inoculated groups (*P* ≥ .05; Figure 2). Thirteen of 14 animals from the coinoculated group had at least 1 of the assessed clinical signs. Individual clinical scores in this group ranged from 0 to 4. In pigs inoculated with swIAV, clinical signs were recorded in 11 of 14 animals and individual clinical scores ranged from 0 to 3.66, whereas in the group inoculated with PRRSV, 10 of 14 piglets had clinical abnormalities and individual clinical scores ranged from 0 to 3. The control pigs did not have any clinical signs.

### 3.2 Pathogen shedding

**FIGURE 3:**
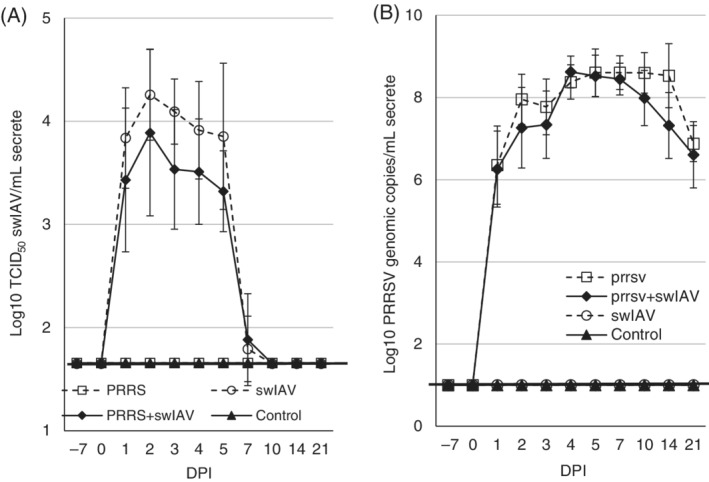
Nasal virus shedding (mean ± SD) after inoculation of pigs with swine influenza A virus (swIAV), A or porcine reproductive and respiratory syndrome virus (PRRSV), B. The dashed line represents the detection limit

The AUC value for SwH1N1 and PRRSV shedding, which was obtained by plotting SwH1N1 titers or PRRSV genomic copies of pigs sampled from day 0 to the last day when the virus was shed (TCID_50_ titer or copy number/mL below the detection limit) vs each time point, did not differ significantly between single inoculated (both viruses) and coinoculated pigs (*P* ≥ .05). The dynamics of shedding of both pathogens (mean ± SD) during study period are presented in Figure 3A (PRRSV) and B (swIAV).

### 3.3 PRRSV in serum

**FIGURE 4:**
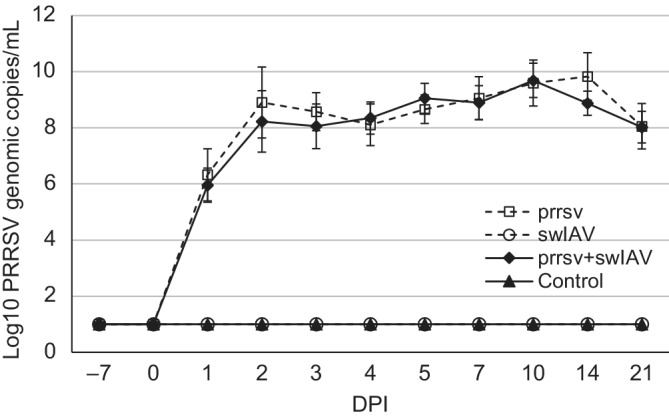
Mean quantitative RT‐PCR results (±SD) on serum samples (PRRSV RNA copies/mL of serum) at each sampling time. The dashed line represents the detection limit

The AUC for PRRSV viremia, which was obtained by plotting genomic copy number against each sampling point, did not differ significantly between pigs single‐inoculated and pigs coinoculated (*P* ≥ .05). The dynamics of PRRSV viremia (mean ± SD) during the study period are presented in Figure 4. The RNA of PRRSV was detected in the serum samples of all PRRSV‐inoculated or coinoculated animals. On average, PRRSV viremia, in both single‐inoculated and coinoculated pigs, started at 1 DPI and lasted until the end of the study (21 DPI). No significant differences were observed between groups inoculated with PRRSV during the entire study period. In control and swIAV only‐inoculated pigs, no PRRSV RNA was found in serum.

### 3.4 Humoral immune response

**FIGURE 5:**
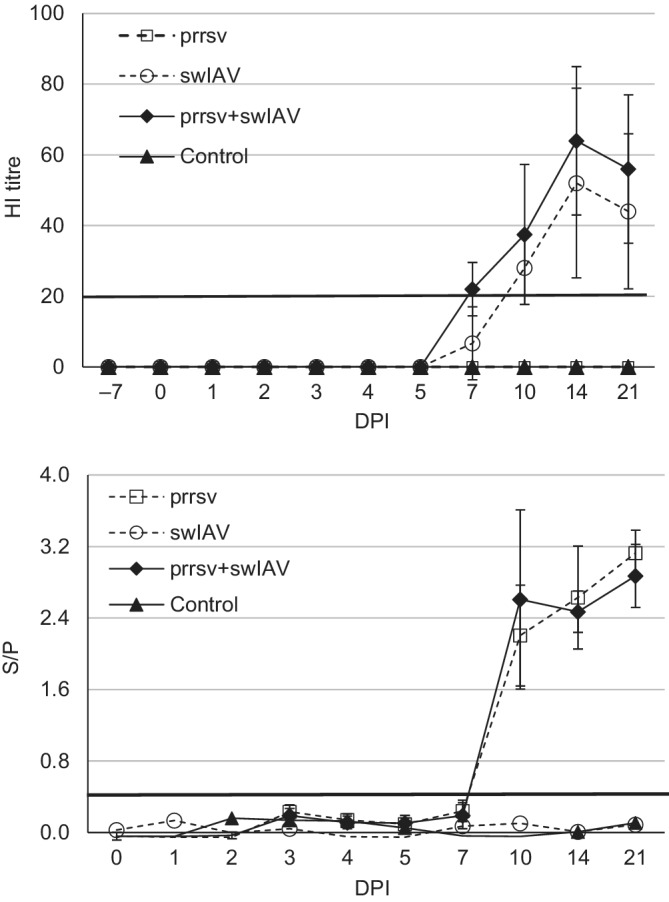
Mean (±SD) HI antibody titers against swIAV in pigs singly or co inoculated with swine influenza A virus (swIAV) or porcine reproductive and respiratory syndrome virus (PRRSV) or both, A and mean (±SD) concentration of antibodies against PRRSV in serum of pigs singly or coinoculated with swIAV or PRRSV or both, B

The humoral response after inoculations of pigs with SwH1N1, PRRSV, or both is presented in Figure 5A,B. Seven of 8 piglets from the coinoculated group seroconverted against swH1N1 at 7 DPI. In pigs from the swIAV group, only 3 of 8 pigs seroconverted at 7 DPI. All coinoculated pigs and those single‐inoculated with swH1N1 showed seroconversion against swH1N1 at 10 DPI. All piglets inoculated with PRRSV or coinoculated with PRRSV + swIAV developed specific antibodies at 10 DPI (with the exception of 1 pig in the PRRSV + swIAV group that seroconverted at 14 DPI). No differences in the magnitude of the humoral response against swIAV and PRRSV between single and coinoculated groups (*P* ≥ .05) were observed.

### 3.5 Cellular immune response

**FIGURE 6:**
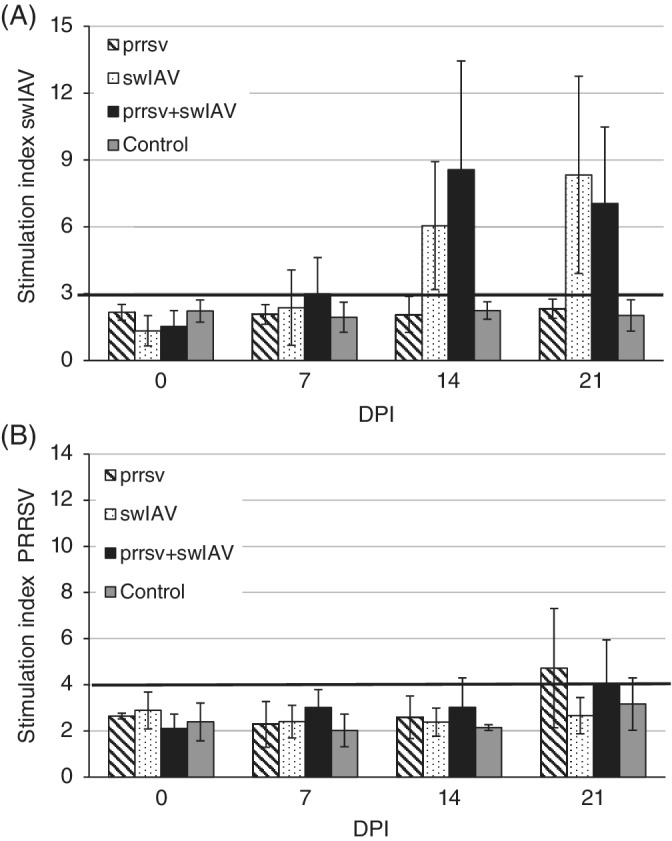
The mean (±SD) value of stimulation index against swine influenza A virus (swIAV) in pigs singly or coinoculated with swIAV or porcine reproductive and respiratory syndrome virus (PRRSV) or both, A and the mean (±SD) value of stimulation index against PRRSV in pigs singly or coinoculated with swIAV or PRRSV or both, B. The bold line indicating value considered as antigen‐specific proliferation

The individual SIx values in control pigs and pigs from experimental groups before inoculation ranged from 1.19 to 3.84 for PRRSV and from 0.97 to 2.85 for swH1N1. Two weeks after inoculation, 1 pig of 5 from the coinoculated group had an individual SIx against PRRSV higher than 4.32. At 21 DPI, 3 of 5 pigs developed an antigen‐specific proliferation against PRRSV. After stimulation of PBMC with swH1N1, an individual SIx value indicating antigen‐specific proliferation in the coinoculated group was observed in 3 of 5 pigs at 7 DPI and in all pigs at 14 and 21 DPI, whereas in single‐inoculated animals at 7 DPI only 1 pig had antigen‐specific proliferation against swH1N1. No significant differences in magnitude of T‐cell response against both pathogens were noted between respective single or coinoculated groups (*P* ≥ .05). Mean SIx values (±SD) against swH1N1 and PRRSV are presented in Figure 6A,B.

### 3.6 Acute phase proteins

**FIGURE 7:**
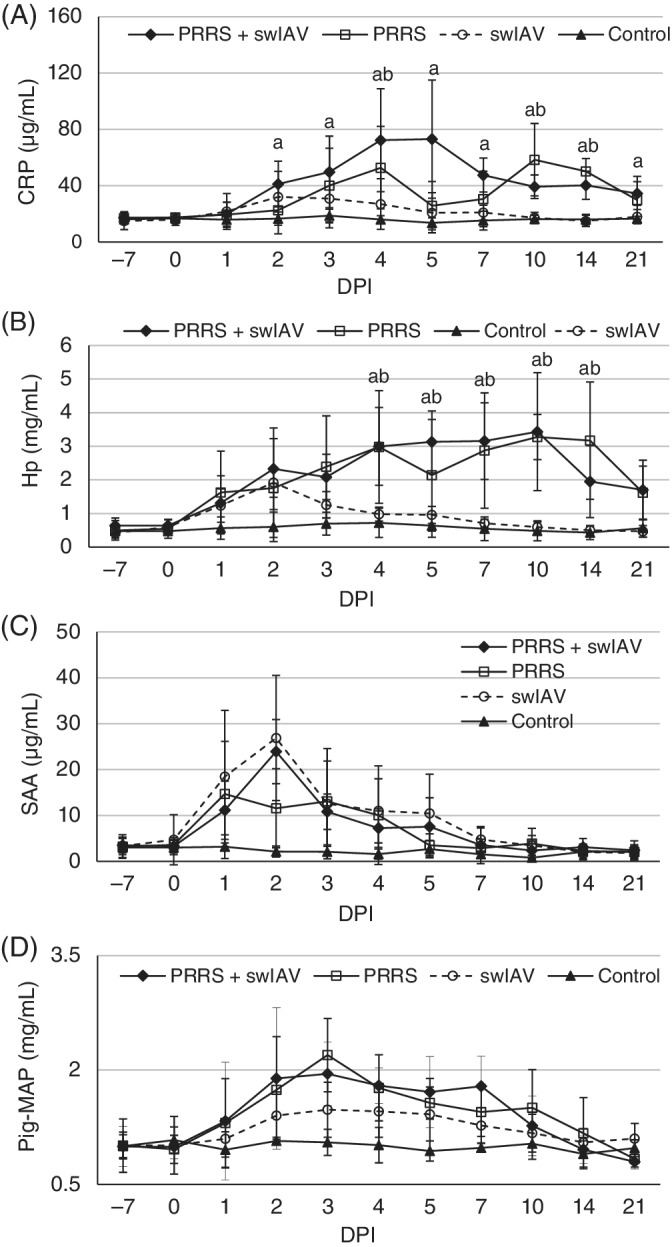
Concentrations of SAA, Pig‐MAP, Hp, and CRP (mean ± SD) in serum of pigs before and on various time point after single or coinoculation with swine influenza A virus (swIAV) or porcine reproductive and respiratory syndrome virus (PRRSV) or both. The significant differences (*P* < .05) within the same day are indicated as follow: a, swIAV + PRRSV group vs control group; b, PRRSV vs control group; c, swIAV vs control group

In the control pigs, serum concentrations of all investigated acute phase proteins were stable during the study period and did not differ significantly from concentrations observed at −7 DPI (Figure 7A‐D).

The serum concentration of CRP increased significantly only in pigs inoculated with PRRSV and in coinoculated pigs (Figure 7A) as compared to controls (*P* < .05). In piglets in the coinoculated group, the mean serum concentration of CRP was significantly increased from 2 DPI until the end of the study (as compared to day 0 concentration and to control animals). In the PRRSV group, different kinetics of serum CRP concentration were noted. The mean serum concentrations of this protein was significantly higher at 4, 10, and 14 DPI as compared to control animals (*P* < .05).

The serum concentration of Hp increased significantly in pigs single or coinoculated with PRRSV as compared to control animals (Figure 7B). The dynamics of serum Hp concentration were similar in both groups inoculated with PRRSV. In piglets from the PRRS + swIAV and PRRSV groups, mean serum concentrations of Hp were significantly increased from 4 to 14 DPI (as compared to day 0 serum concentrations and to control animals).

The serum concentrations of SAA were significantly increased from 2 to 4 DPI in groups inoculated with SIV and PRRSV + swIAV as compared to control pigs (*P* < .05). No significant differences were found between control and PRRSV as well as swIAV and PRRSV + swIAV groups (*P* ≥ .05). Starting from 5 DPI the serum concentrations of SAA in inoculated groups did not differ significantly from those of control animals (*P* ≥ .05; Figure 7C).

The serum concentration of Pig‐MAP remained unchanged as compared to its preinoculation concentration (*P* ≥ .05) in pigs inoculated with SwH1N1 and in control animals (Figure 7D). In piglets from the PRRSV and swIAV + PRRSV groups, significant increases were observed from 3 DPI. The serum concentration of Pig‐MAP remained increased in both groups until 7 DPI as compared to the day 0 concentration and to concentrations in control pigs (*P* < .05). No differences were found between pigs singly and coinoculated with PRRSV (*P* ≥ .05).

### 3.7 Lung lesions and pathogen load

**FIGURE 8:**
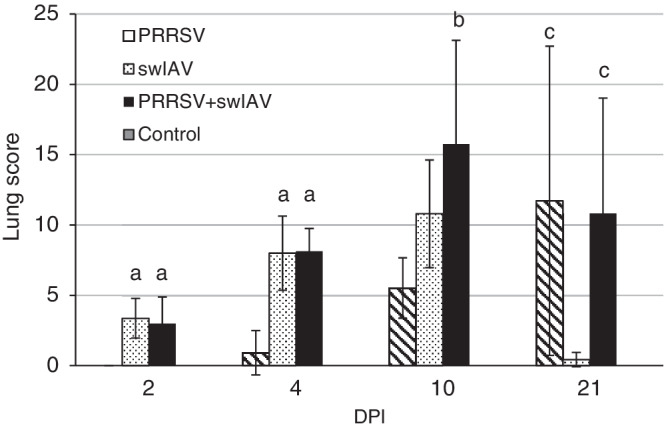
Lung lesion scores (mean ± SD) observed in pigs singly or coinoculated with swine influenza A virus (swIAV) or porcine reproductive and respiratory syndrome virus (PRRSV) or both. a, significant differences between swIAV and PRRSV + swIAV group vs PRRSV and control group (*P* < .05); b, significant differences PRRSV + swIAV group vs PRRSV and control group (*P* < .05); c, significant differences between PRRSV and PRRSV + swIAV group vs swIAV and control group (*P* < .05)

Lung lesions characteristic for viral infection of variable severity (LS 1 to 15 in swIAV, 0 to 28 in PRRSV, and 1.25 to 23.5 in swIAV + PRRSV) were observed in all pigs inoculated or coinoculated with swIAV from 2 to 10 DPI. At 21 DPI, no pathological lesions were found in 3 of 5 swIAV single‐inoculated animals. In PRRSV single‐inoculated pigs, no lesions were observed at 2 and 4 DPI, whereas at 10 and 21 DPI lung lesions typical of PRRSV infection were found in all pigs. Control pigs did not show any pathological lesions. At 2 and 4 DPI, mean LS noted in the swIAV and PRRSV + swIAV groups were significantly higher than those in the PRRSV and control groups (*P* < .05). At 10 DPI, the mean LS in the coinoculated pigs was higher than in the PRRSV and control groups (*P* < .05). Mean LS differed significantly at 21 DPI between pigs singly or coinoculated with PRRSV and controls (*P* < .05), as well as compared to swIAV single‐inoculated pigs (*P* < .05). The mean LS observed in all experimental groups are presented in Figure 8.

**FIGURE 9:**
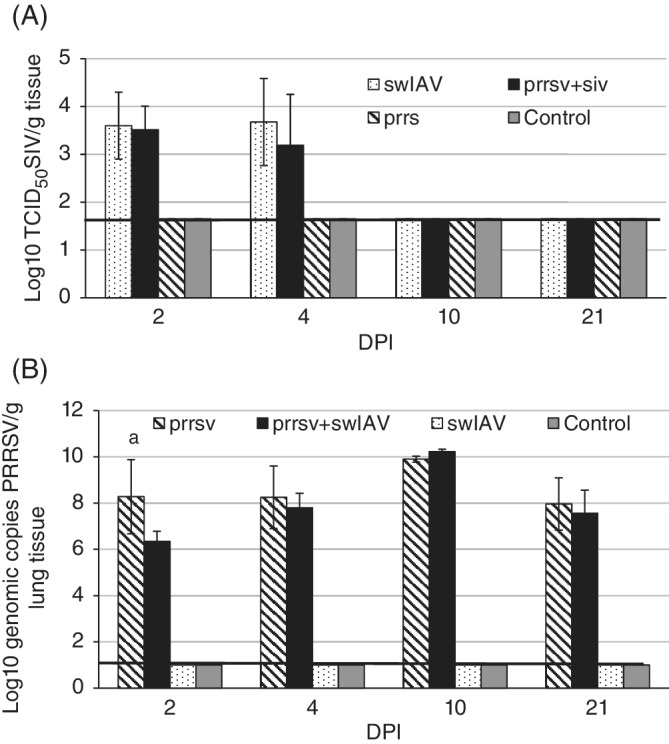
Swine influenza A virus (swIAV) titers, A and the mean copy number of porcine reproductive and respiratory syndrome virus (PRRSV), B, (mean ± SD) in the lung at 2, 4, 10, and 21 days after single or coinoculation of pigs. The dashed line represents the detection limit. a, significant differences between PRRSV and PRRS + swIAV group (*P* < .05)

The RNA of swIAV was detected in all samples taken from the right lungs of pigs inoculated with swIAV at 2 and 4 DPI. At 10 and 21 DPI, no swIAV was detected in any of the groups. The mean swIAV TCID_50_ titers did not differ significantly between pigs singly‐inoculated and coinoculated with swIAV (*P* ≥ .05). In contrast, significant differences were found between mean copy number of PRRSV in lungs taken from pigs in the PRRSV and PRRSV + swIAV groups (*P* < .05) at 2 DPI, which was significantly higher in PRRSV only‐inoculated pigs. At 4, 10 and 21 DPI, no significant differences in lung PRRSV load were observed (*P* ≥ .05). The PRRSV was detected in samples taken from right lung samples of all PRRSV singly and coinoculated pigs from 2 to 21 DPI. Virus loads in the lungs at 2, 4, 10, and 21 DPI are presented in Figure 9A,B.

## 4 DISCUSSION

The viruses PRRSV and swIAV, alone or in combination, are 2 important pathogens among viruses contributing to porcine respiratory infections.^5^, ^9^, ^10^ The PRRSV predisposes pigs to coinfection by other respiratory viruses, because of destruction of pulmonary tissues.^18^, ^21^, ^28^, ^29^ Additionally, immunosuppression induced by PRRSV may enhance the severity of other respiratory viral coinfections^20^, ^21^ and decrease the efficacy of immunization, including vaccination against swIAV.^13^ Previous studies indicated that pigs infected with PRRSV were more likely to be coinfected with swIAV and developed 11%‐50% higher LS. Moreover, PRRSV‐infected pigs at the ages of 9 and 16 weeks were 15.57 and 5.75 times more prone to swIAV coinfection.^4^ Despite the marked economic importance of those viruses and frequent coinfections observed in the field, relatively few studies have explored their possible interactions. Moreover, most of the in vivo animal experiments were performed many years ago with early isolates of these rapidly‐evolving viruses and results reported were somewhat inconsistent and did not provide a complete picture of the effects of coinfection.

We assessed the impact of coinfection vs single infections with swIAV or PRRSV on the clinical characteristics, immune and acute phase responses, viral shedding and viral load. Viral strains recently circulating in Poland were used in the study.

Our results show that simultaneous infection with PRRSV and swIAV had limited impact on the clinical outcome and immune response. For most studied variables, no significant differences were observed between pigs coinfected and singly infected with PRRSV and swIAV, including clinical scoring. Although clinical scores between groups were not statistically different, the highest number of pigs with persistent fever and longest duration of fever were observed in the coinfected group. Previous studies on PRRSV and swIAV coinfections produced some conflicting results regarding impact on the clinical course. One study reported more pronounced clinical signs, including fever, respiratory lesions and growth retardation in pigs infected with PRRSV and coinfected with swIAV 3 days later.^20^ Another study found significant enhancement of clinical respiratory lesions and PRRSV‐related interstitial pneumonia in pigs simultaneously infected with swIAV and PRRSV at 7 weeks of age.^13^ In another study, in which piglets were infected intranasally with PRRSV, followed 1 week later with H3N2 swIAV strain, no influence on the clinical course of influenza infection was detected.^7^ Another study observed variable clinical outcomes, depending on both the time interval between infections and the health status of pigs used in the study.^6^ Another factor increasing variability among different studies may be the inherent virulence of viral strains used, especially PRRSV. One groups of investigators infected pigs with PRRSV‐2, considered to be more virulent than PRRSV‐1 used in other studies.^13^

Another study determined that swIAV replication was slightly affected by prior infection with PRRSV, and viral excretion in the PRRSV‐swIAV group was delayed by 2 days, not only with regard to presence of the virus, but also with respect to the peak amount.^20^ We did not observe any delay or decrease in swIAV shedding in the coinoculated group compared to single‐inoculated animals. In contrast, a short‐lived significant decrease of PRRSV replication in the lung was found at 2 DPI in coinoculated pigs compared to the single‐inoculated PRRSV group (*P* < .05).

The limited impact of coinfection on clinical outcome, viral load, and shedding may be a result of differences in cellular targeting between the 2 viruses. Differentiated macrophages, mainly pulmonary alveolar macrophages (PAMs) and pulmonary intravascular macrophages, are the primary target for PRRSV. The virus also may replicate, to a lesser extent, in dendritic cells and monocyte‐derived macrophages present in most organs.^30^ The swIAV infects epithelium of the respiratory tract (bronchi, bronchioles and alveoli) and replicates extensively in porcine lungs.^31^ Porcine respiratory coronavirus (PRCV), which also replicates mainly in the epithelium of the lower respiratory tract, strongly interfered with swIAV infection, decreasing swIAV replication by 99%.^32^ On the other hand, PRRSV and PRCV coinfected pigs exhibited more severe clinical and lung lesions, but no impact on replication level and virus shedding was observed.^20^

Although a previous study that used a recombinant cell line susceptible to infection by both viruses, confirmed that the viruses were interfering with each other,^33^ in a natural host only a small population of dendritic cells and in some circumstances type 1 pneumocytes can be targeted by both PRRSV and swIAV. The interference of swIAV with PRRSV numbers at 2 DPI could be the result of increased amounts of interferon alfa (IFN‐α) produced by plasmacytoid dendritic cells (pDCs), and other types of cells, in response to swIAV infection. The effect of PRRSV replication inhibition previously was observed in vitro after coinfection of PAMs by an IFN‐α‐stimulating PCV2 strain.^34^ Although in the case of swIAV and PRRSV the possibility of coinfecting the same cells is limited, such interaction could have an indirect effect. One study detected an increased concentration of IFN‐α in bronchoalveolar lavage fluid (BALF) of swIAV‐infected pigs at 3 DPI, which coincides with the decrease in of PRRSV concentration in coinfected pigs in our study.^35^

At 2 DPI, when a significant decrease in PRRSV replication was detected in the lungs of coinfected pigs, no visible lung lesions were detected in the PRRSV‐infected group. Simultaneously, LS in the swIAV only and coinfected groups were comparable, indicating that within this time frame swIAV was the sole cause of pathological lesions in the lungs. Differences in LS between experimental groups (singly or coinfected) observed later in our study most likely were related to the dynamics of infection with the viruses (swIAV or PRRSV) and not to interactions between them. In the case of swIAV, lung lesions appeared earlier and their decrease coincided with the most intensive development of PRRSV‐associated lung lesions. Analysis of the LS pattern over time in the coinfected group indicated that LS reflected the additive dynamics of swIAV only‐ and PRRSV only‐infected groups. The highest LS was recorded in the coinfected group 10 DPI and significantly exceeded that of the PRRSV group. Mean LS at this time also was higher, however not significantly, compared to the swIAV inoculated group. Most probably, the mild increase in severity of clinical and microscopic lesions, also reported in previous studies, is an effect of increased damage of lung structure and inflammation in dual infection.^13^, ^20^ Infection with swIAV causes epithelial cells necrosis, increased proinflammatory mediator production and infiltration with phagocytic cells susceptible to PRRSV infection.^35^, ^36^ Respiratory signs caused by PRRSV also are the result of pathology in the lung and interaction with the host immune system. The virus causes interstitial pneumonia and induces TNF‐α‐mediated apoptosis, also in noninfected bystander macrophages.^37^ Because our study was performed under strictly controlled conditions in a BSL3 facility, it does not correspond to field conditions, where many factors may play a role. Specifically, different amounts of bacterial infection in the respiratory tract may interfere with the clinical and pathological course of viral coinfections and contribute to outcome, often resulting in PRDC.

Our results also show that coinfection with local PRRSV and swIAV strains did not affect the intensity and kinetics of the acute phase and immune responses. The results of a previous study identified that the acute phase response was markedly different between strains in terms of intensity and duration,^38^ but Hp was the most sensitive biomarker for PRRSV infection. In addition, Hp and CRP discriminated between infected and control pigs. This finding is in agreement with our results, because the significant increase in Hp and CRP was noted only in groups inoculated or coinoculated with PRRSV. In addition, significant differences in Hp between PRRSV and PRRSV + swIAV groups and the swIAV group were observed from 4 to 14 DPI. No significant differences in magnitude of PBMC proliferation and humoral response against both pathogens were noted between respective singly or coinoculated groups (*P* ≥ .05). In contrast, another study observed that PRRSV increased swIAV‐specific lymphocyte proliferation in PBMCs collected 4 weeks after coinfection, but the PRRSV‐2 strain was used. ^13^ Few effects on the innate immune response after coinfection with swIAV and PRRSV also were observed in a previous study conducted on conventional pigs.^39^ The investigators concluded that coinfection with PRRSV and swIAV has additive effects only on the mRNA expression of interleukin (IL) 6 and IL‐10, among 6 investigated cytokines (IL‐1β, IL‐6, IFN‐γ IL‐8, IL‐10, and IFN‐α), but the impact of such synergy on viral load and severity of clinical disease is not clear and requires further investigation. An additional study examined coinfections with swIAV and PRRSV in vitro and ex vivo^40^ and found synergy for some specific targets such as toll like receptor 3 (TLR3), retinoic acid‐inducible gene I (RIG‐I), and interferon beta (IFNβ) transcripts in precision‐cut lung slices (PCLS) when the viruses were administered concomitantly. The investigators concluded that the impact of such synergy on clinical outcome is difficult to establish because it can either increase clinical signs and be detrimental for the host or may assist in the rapid clearance of the infections.

Although PRRSV may be immunosuppressive and act synergistically with some pathogens, simultaneous coinfection of pigs with swIAV and PRRSV did not potentiate the severity of clinical signs, lung lesions, immune response and replication of both viruses in the respiratory tract. The absence of synergy between the 2 viruses after their replication is beneficial for the host, because it should not lead to worsening of lung changes and clinical signs, despite common coinfection under field conditions.

## CONFLICT OF INTEREST DECLARATION

Authors declare no conflict of interest.

## OFF‐LABEL ANTIMICROBIAL DECLARATION

Authors declare no off‐label use of antimicrobials.

## INSTITUTIONAL ANIMAL CARE AND USE COMMITTEE (IACUC) OR OTHER APPROVAL DECLARATION

Animal use and handling protocols were approved by II Local Ethical Commission for the Animal Experiments of University of Life Sciences in Lublin (number of approval: 77/2014).

## HUMAN ETHICS APPROVAL DECLARATION

Authors declare human ethics approval was not needed for this study.

## Data Availability

The data that support the findings of this study are available from the corresponding author upon reasonable request.
